# Staff training to improve participant recruitment into surgical randomised controlled trials: A feasibility study within a trial (SWAT) across four host trials simultaneously

**DOI:** 10.1177/26320843221106950

**Published:** 2022-06-15

**Authors:** Adwoa Parker, Catherine Arundel, Nicola Mills, Leila Rooshenas, Marcus Jepson, Jenny L Donovan, Jane M Blazeby, Elizabeth Coleman, Laura Clark, Laura Doherty, Catherine E Hewitt, Prasanna Partha Sarathy, David Beard, Peter Bower, Stephen Brealey, Paul Brocklehurst, Cindy Cooper, Julie Croft, Lucy Culliford, Joseph Dias, Declan Devane, Sandra Eldridge, Richard Emsley, Sandra Galvin, Elke Gemperle-Mannion, David G Jayne, Andrew J Metcalfe, Alan Montgomery, Amar Rangan, Christopher J Sutton, Puvanendran Tharmanathan, Shaun Treweek, David Torgerson

**Affiliations:** 1 8748 York Trials Unit, The University of York, York, North Yorkshire, UK; 2 1980 University of Bristol, Bristol, UK; 3 8749 York Teaching Hospital NHS Foundation Trust, York, North Yorkshire, UK; 4 6396 University of Oxford, Oxford, UK; 5 5292 University of Manchester, Manchester, UK; 6 1506 Bangor University, Bangor, UK; 7 7315 The University of Sheffield, Sheffield, UK; 8 4468 University of Leeds, Leeds, UK; 9 4488 University Hospitals of Leicester NHS Trust, Leicester, UK; 10 8799 National University of Ireland Galway and Health Research Board-Trials Methodology Research Network, Republic of Ireland; 11 4617 Queen Mary University of London, London, UK; 12 4616 King’s College London, London, UK; 13 2707 University of Warwick, Coventry, UK; 14 6123 The University of Nottingham, Nottingham, UK; 15 1019 University of Aberdeen, Aberdeen, UK

**Keywords:** randomised controlled trial, study within a trial, recruitment, staff training, professional education, feasibility study, surgical trials

## Abstract

**Objective:**

To test the feasibility of undertaking a simultaneous Study Within A Trial (SWAT) to train staff who recruit participants into surgical randomised controlled trials (RCTs), by assessing key uncertainties around recruitment, randomisation, intervention delivery and data collection.

**Study design and setting:**

Twelve surgical RCTs were eligible. Interested sites (clusters) were randomised 1:1, with recruiting staff (surgeons and nurses) offered training or no training. The primary outcome was the feasibility of recruiting sites across multiple surgical trials simultaneously. Secondary outcomes included numbers/types of staff enrolled, attendance at training, training acceptability, confidence in recruiting and participant recruitment rates six months later.

**Results:**

Four RCTs (33%) comprising 91 sites participated. Of these, 29 sites agreed to participate (32%) and were randomised to intervention (15 sites, 29 staff) or control (14 sites, 29 staff). Research nurses attended and found the training to be acceptable; no surgeons attended. In the intervention group, there was evidence of increased confidence when pre- and post- training scores were compared (mean difference in change 1.42; 95% CI 0.56, 2.27; *p* = 0.002). There was no effect on recruitment rate.

**Conclusion:**

It was feasible to randomise sites across four surgical RCTs in a simultaneous SWAT design. However, as small numbers of trials and sites participated, and no surgeons attended training, strategies to improve these aspects are needed for future evaluations.

## Introduction

Randomised controlled trials (RCTs) are the gold standard for investigating the efficacy, effectiveness and safety of health-care interventions.^1^ A major challenge faced by RCTs is recruitment: approximately 50% of trials fail to recruit sufficient numbers of participants.^2^ Low recruitment rates can be costly, wasteful, lead to sampling bias and reduce statistical power.^3, 4^ It can also lead to delays in the adoption of effective treatments and the continued use of ineffective or even harmful treatments to patients. Recruitment to RCTs is dependent on patient and staff factors.^5^ Trials evaluating surgical interventions are especially challenging as both patients and recruiting staff may have a strong preference for one of the study treatment arms.^6^ These factors lead to one third of surgical trials closing prematurely or findings from completed trials going unpublished.^7^

In recognising and trying to prevent low recruitment rates in RCTs, a growing evidence base has identified, developed and tested recruitment strategies and methods. Evaluation of strategies is often done by nesting or embedding research into the main host trial; often referred to as a study within a trial (SWAT). SWATs are frequently evaluated in a single trial; meaning there can be limited statistical power to detect the small effects (e.g. <5% change) that are typical with such interventions. Therefore, evaluations in multiple trials are required, with pooled estimates of effect through meta-analyses where possible. Undertaking SWATs in an uncoordinated, opportunistic way means that it can take many years to derive sufficient evidence to facilitate a meta-analysis. An alternative approach is to coordinate SWATs simultaneously across several trial settings; the benefit is that a definitive answer can be reached more quickly. If the strategy is effective, this can therefore be rapidly implemented into trial settings, accordingly, thus potentially limiting slow trial recruitment and so increasing trial efficiency.^8^

The most recent Cochrane recruitment review identified only three strategies that had a high certainty evidence that the intervention increased recruitment rates; all three are focused on research participants. Five RCTs in the Cochrane review investigated strategies targeted at staff; however, only one of these investigated staff training.^9^ The training of trial recruiters has been identified as the top priority topic for recruitment research according to the Directors of UK Clinical Trials Units^10^ and has been highlighted in a James Lind Alliance priority setting exercise for recruitment research.^11^ A systematic review of recruiter training showed that training programmes were well received and increased recruiters’ self-confidence. However, only three of the 17 studies included were RCTs and, when assessed using the Effective Public Health Practice Quality Assessment tool,^12^ most were of moderate or weak quality; there was also little evidence that training increased actual recruitment rates.^13^ A more recent systematic review looking at the effectiveness of staff training on recruitment concluded that further work on developing a substantial evidence base around the effectiveness of education and training interventions for recruiters to trials is required.^14^ As a result, there is a lack of evidence on the effectiveness of staff training to improve recruitment.

Given the limited evidence available, we aimed to assess the feasibility of undertaking a Study Within A Trial (SWAT) of a recruiter training intervention into multiple surgical trials simultaneously. Our objective was to assess key uncertainties around recruitment, randomisation, intervention delivery and data collection. A secondary objective was to assess trainee confidence and participant recruitment.

## Methods

### Trial design

A parallel, feasibility cluster-randomised SWAT, undertaken in multiple host trials simultaneously.

### Host trial and participant recruitment

To be eligible, host trials had to undertake face-to-face recruitment (instead of recruiting exclusively using postal, online or telephone-based methods). Eighteen Clinical Trial Units (CTUs) delivering surgical trials across the United Kingdom were contacted, and surgical RCTs recruiting or likely to be recruiting in April 2019 were invited to take part in this SWAT. This included all seven Royal College of Surgeons Surgical Trials Centres.^15^ We met with the UK NIHR Clinical Research Network (including the Lead for Surgical Trials), which supports recruitment into research in the UK. They identified two additional potential host surgical trials that would be eligible for the SWAT. Once a trial team expressed an interest, staff at recruiting sites within the host trials were contacted by the host trial team, using a standardised invitation, to assess their interest in attending a training workshop. Upon expressing willingness to participate in the training, recruiting sites within each host trial were entered in the SWAT.

### Intervention

The University of Bristol’s QuinteT team (Qualitative research integrated within Trials) has developed a one-day training course for staff, which was used as the intervention for this study. Developed through the MRC Hubs for Trials Methodology Research and the ConDuCT-II Hub, this training has previously been delivered to 99 health professionals (67 surgeons and 32 research nurses) and has been shown to improve confidence in recruitment.^16^ The training aims to share experiences, raise awareness of the hidden challenges of recruitment and equip attendees with strategies to optimise recruitment and informed consent. The material covered in the workshop was empirically based, addressing the clear obstacles and hidden challenges of recruitment identified from a synthesis of QuinteT Recruitment Intervention (QRI) studies embedded within six pragmatic trials and supplemented with findings from related studies, across a range of clinical settings.^17-20^ The topics covered are outlined in Box 1.

• Hidden challenges from screening to approaching patients

o Recruitment as a complex process

o Pathway challenges

o Determining eligibility

o Approaching patients

• Hidden challenges within recruitment discussions

o Conveying equipoise

o Engaging with treatment preferences

o Considering RCT terminology

Box 1. Topics covered on the recruitment training course.

All staff members involved in recruitment (surgeons, research nurses and allied health professionals) were invited to attend a one-day training course relevant to their profession (one date was offered to surgeons, and one to research nurses and allied health professionals such as physiotherapists or hand therapists). To facilitate attendance, the training workshop was organised in a central location in the United Kingdom (Birmingham), with participants being offered travel expenses and accommodation if required. Academic researchers within the University of Bristol’s QuinteT group delivered the training.

The one-day training was supplemented with the GRANULE (Generating surgical recruiters to randomised trials) online e-learning course (https://learn.nihr.ac.uk/course/view.php?id=385). The course is a collaboration between The Universities of Birmingham and Bristol and hosted on the NIHR Learn Platform,^21^ designed to equip recruiters with the practical skills to discuss RCT recruitment with surgical patients. This online training, also delivered by members of the QuinteT team, presents a reduced version of the face-to-face course delivered in the present study, focusing primarily on the key hidden challenges with recruitment discussions. All staff from the sites randomised to the intervention arm were offered the online training. Staff who attended the face-to-face training were sent the link to the GRANULE training after attending the training to promote their learning. Staff in the intervention sites who could not attend the training were also sent the link to GRANULE, along with the summary sheet. All staff in the intervention sites were encouraged to share the GRANULE link and summary sheet to other colleagues recruiting participants at their sites. Appendix 1 outlines the intervention, using the ‘Template for Intervention Description and Replication’ (TIDieR) checklist.^22^

### Outcomes

In this feasibility study, the primary outcome of interest was the ability to recruit multiple surgical trials simultaneously. To be considered feasible, we needed to enrol a minimum of two host trials. Secondary outcomes of interest were:

1. Recruitment: Numbers of recruiting sites and recruiting staff from each site enrolling into the SWAT
2. Randomisation: Number of sites recruited, number of sites randomised and any reasons for sites dropping out after recruitment.
3. Intervention delivery: number of intervention training course groups initiated, staff attendance at training in the intervention group, number of participants per group, any reasons for non-attendance or failures in intervention delivery.
4. Acceptability of the training
5. Staff confidence in discussing trial recruitment with potential participants immediately before, immediately after, and at 1–3 months post-training
6. Participant screening and recruitment rate (defined as the proportion of eligible participants who gave their consent and were randomised into the host trial six months following delivery of the course).

### Randomisation and blinding

Randomisation was undertaken by a statistician independent of both the delivery of the host trials and SWAT training interventions. Randomisation was performed separately for each host trial at the cluster (i.e., recruiting hospital site) level. On expressing willingness to participate in the training, recruiting sites within each host trial were randomised to be offered the training workshop (intervention group) or no training (usual recruitment practice=control group) on a 1:1 basis. A computer-generated randomisation schedule was used, generated using permuted blocks of size 2, 4 and 6, stratified by recruiting trial. Research participants (i.e., host trial recruiting staff) involved in the host trials were blind to the SWAT hypothesis, but the sites could not be blinded to the intervention.

### Data collection

Online questionnaires were distributed to participants using the Qualtrics platform (QualtricsXM, Provo, UT, USA). Two reminders were sent along with each questionnaire to increase response rates.

Participants were asked to complete questionnaires one month before the workshop (all participants), immediately after the workshop (intervention group only) and 1–3 months after the training (all participants).

A flexible approach to questionnaire completion at 1–3 months was employed to allow the participants to have the chance to approach and attempt to recruit patients and collect the follow-up questionnaire once from all participants. Participants were approached to complete the post-training questionnaire after one month; should they not have had the chance to recruit a patient, they were contacted again at two months. At three months all participants who had yet to respond were asked to complete the post-training questionnaire regardless of whether they had approached patients since the workshop.

All the participants were assigned a unique study number to have paired data for pre- and post-training questionnaires. A summary of data collected at each time point and the questionnaires used is available in Appendix 2. The self-confidence questions are based on the work of Jenkins et al^23^ to evaluate a training intervention for those recruiting to cancer RCTs; they were modified to be made relevant for surgical RCTs by Mills et al^16^ and adapted for this SWAT.

### Statistical analysis

All statistical analyses were undertaken in Stata v15, using an intention to treat basis, and a 5% significance level. Baseline characteristics are reported descriptively by trial arm, using counts and percentages for categorical data, and mean and standard deviation for continuous data. Paired t-tests were used to compare pre- and post-training workshop responses. The average score for the self-confidence questions at baseline, post training (intervention arm only) and follow-up are reported as mean and standard deviation by arm. A linear regression model adjusting for baseline score and allocation was run on each of the 11 questions to compare the self-confidence at follow-up between the arms. The results are presented as the coefficient, with associated 95% confidence interval (CI) and *p*-value. The recruitment rate and the number of patients screened over the six months post-training were compared at the site level using linear regression adjusting for host trial and SWAT intervention. Due to large differences in the number of patients screened and recruited at each site, the model was rerun also adjusting for the number of patients screened at site in the month before training being delivered. There was not enough data to explore any difference between face-to-face intervention and the online training.

## Results

### Recruitment of RCTs, sites and participants

Between October 2018 and April 2019 we approached 18 Clinical Trials Units (CTU) delivering surgical trials and identified 13 trials, 12 of which were eligible to participate in the SWAT. Eight (67%) trials declined to participate for a variety of reasons including the limited number of workshop dates offered, not being at the right time for the host trial and plans to engage with the QuinteT training at a later stage. One potential trial was excluded as it was not evaluating a surgical intervention.

Four trials (involving a maximum of 91 recruitment sites) agreed to participate in this SWAT; details on the trials can be found in Table 1.

**Table 1.** Details on the RCTs included in this SWAT.

Trial	Area	Interventions	Number of sites	Target sample size	Primary outcome (timepoint)	Trial registration
DISC	Dupuytren’s contracture in adults over 18	Injection of collagenase or surgery (≥18 years old)	30	710	Patient evaluation measure (2years)	ISRCTN18254597
IntAct	Rectal cancer in adults over 18	Intra-operative fluorescence angiography (IFA), or white light endoscopic surgery	25 (14 UK)	880	Clinical anastomotic leak rate within 90 days post-operation (1 year)	ISRCTN13334746
PROFHER-2	Acute 3- and 4-part fractures of the proximal humerus in patients aged over 65 years	Reverse shoulder arthroplasty, hemiarthroplasty or non-surgical treatment	40	380	Oxford shoulder score (2 years)	ISRCTN76296703
START: REACTS	Rotator cuff tears that cannot be repaired	Arthroscopic debridement or arthroscopic debridement with the InSpace balloon	16	212	Constant-murley score collected 12 months after surgery	ISRCTN17825590

### Feasibility of recruiting host trials

We found recruitment of surgical host trials to occur as planned and were able to recruit four host trials in a six-month period. The QuinteT recruitment work was well known to the host trial teams, and in our communications with host trials, most teams indicated clearly whether they felt the training would be suitable for their individual trials. It is likely we would have recruited two additional host trials if they had not already planned to engage with the QuinteT work later on, or were actively in the process of implementing qualitative research to support recruitment, in a similar way to the QuinteT model. One declining trial said that they were experiencing recruitment difficulties, and were planning recruitment training events for all their sites around the same time as our training intervention was to be delivered; they were therefore not willing to risk randomising half of their sites to not receive additional recruitment training.

### Feasibility of recruiting sites, participants and randomisation

**Figure 1.**
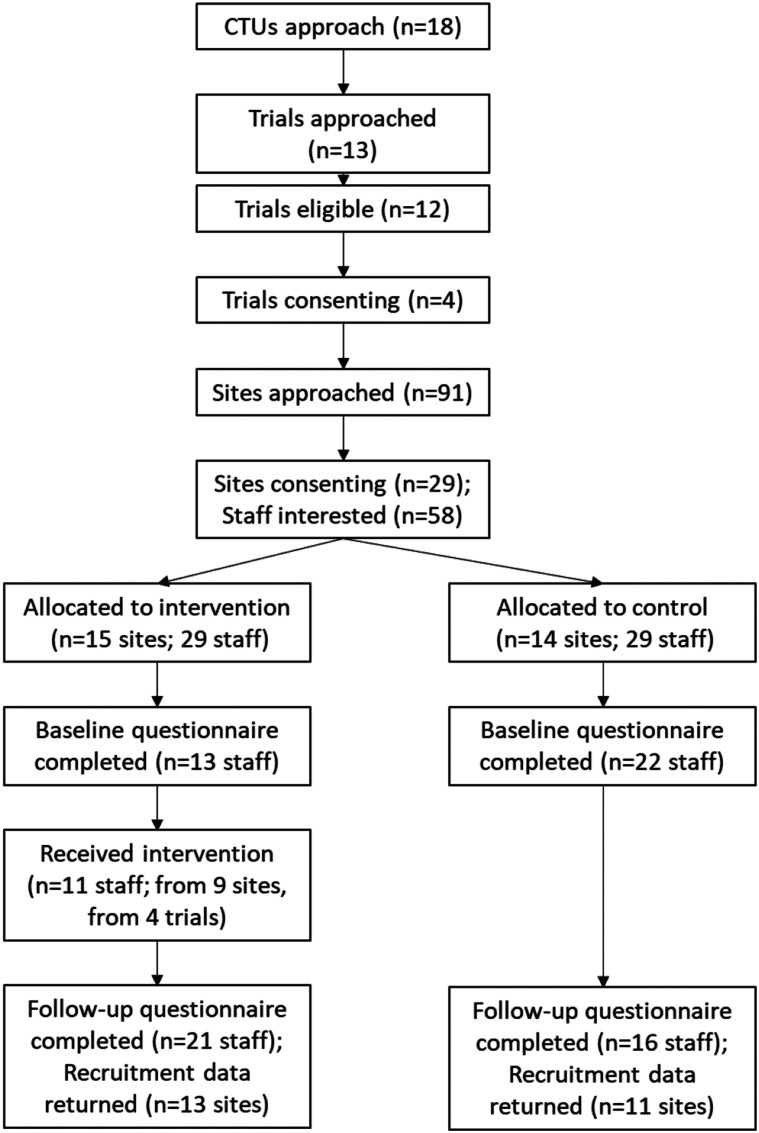
Flowchart of host trial and staff recruitment into the SWAT.

Twenty-nine recruiting sites expressed an interest; all agreed to participate and were randomised into the SWAT: six sites from DISC (20% of sites approached: three intervention, three control); five sites from IntAct (25% of sites approached: three intervention, two control); 15 sites from PROFHER-2 (52% of sites approached: eight intervention, seven control); and three sites from START: REACTS (25% of sites approached: two intervention, one control). A flowchart of the recruitment process is shown in Figure 1.

Of the 29 sites, 14 were randomised to the control group and 15 to the intervention group. Fifty-eight members of staff expressed an interest in taking part in the SWAT from these sites: 29 (50%) from control sites, and 29 from intervention sites (50%). Approximately 37.4% of recruiting staff (58/155) across all sites expressed an interest in participating in the SWAT and were randomised. An average of two recruiting staff per site expressed an interest in participating in the SWAT; ranging from one to six recruiting staff depending on the recruiting site’s size.

We found it feasible to recruit sites and participants. However, not all sites that enrolled were actively recruiting participants into the host trials, and 5 sites were in the set-up stage; one started recruiting participants one month after the training, and three sites did not start recruiting patients until significantly after they enrolled in the SWAT - one in September 2019 and two in 2020. We also found it feasible to randomise sites. All recruited sites were randomised.

### Baseline characteristics

Of the 58 recruiting staff enrolled and randomised, basic demographic information was received for all; and 35 (60.3%) completed the baseline questionnaire following randomisation. Seventeen (47.2%) of the recruiting staff indicated at baseline that they had been actively involved with recruiting participants to the PROFHER-2 trial; nine (25.0%) on IntAct; eight (22.2%) on DISC; and eight (22.2%) on START:REACTS. Some recruiting sites worked across multiple trials involved in this SWAT. Table 2 outlines the characteristics of staff who expressed an interest in the training.

**Table 2.** Characteristics of the members of staff who expressed an interest in attending the training.

Characteristics	Control (*n*=29)	Intervention (*n*=29)
**Gender**		
Male	13 (44.8)	8 (27.6)
Female	16 (55.2)	21 (72.4)
**Age in years**		
20-29	1 (3.5)	3 (10.3)
30–39	2 (6.9)	3 (10.3)
40-49	12 (41.4)	5 (17.2)
50–59	7 (24.1)	2 (6.9)
>60	0 (0.0)	1 (3.5)
Missing	7 (24.1)	15 (51.7)
**Role**		
Surgeon	9 (31.0)	9 (31.0)
Research nurse	17 (58.6)	12 (41.4)
Other	2 (6.9)	5 (17.2)
Missing	1 (3.5)	3 (10.3)
**No. Of years recruiting to rcts**		
<1	6 (20.7)	4 (13.8)
1–2	5 (17.2)	4 (13.8)
3-5	5 (17.2)	5 (17.2)
>5	6 (20.7)	1 (3.4)
missing	7 (24.1)	15 (51.7)
**Total No of RCTs involved in**		
*N*, mean (SD.)	22, 4.5 (3.2)	14, 4.4 (4.0)
**Previous training in recruiting**		
Yes	14 (48.3)	9 (31.0)
No	8 (27.6)	5 (17.2)
Missing	7 (24.1)	15 (51.7)

The recruiting staff were mostly research nurses (58.6% control and 41.4% intervention) and surgeons (31.0% in both groups). Most recruiting staff had previously received some training in recruiting patients formally or informally (63.6% control and 64.3% intervention). Recruiting staff reported working across four to five different RCTs simultaneously to recruit participants (average of 4.5 and 4.4 for the control and intervention, respectively). Participants in the intervention group were predominantly female (72.6% compared to 55.2% in the control group) and 20.6% of recruiting staff below the age of 40 compared to 10.3% in the control group.

### Feasibility of training course and acceptability

Of the 29 participants interested in attending the workshop, 11 (37.9%) attended. Those who attended the workshop were all female (100%), predominately research nurses (81.8%) and the modal age was 40–49 years (45.5%). Only one surgeon agreed to attend the workshop, which resulted in the surgeons’ workshop being cancelled. While other surgeons were interested, clinical and personal commitments prevented them from being able to attend on the date given. One surgeon who sent their apologies was from a site that did not start recruiting patients until 2020, so it may not have been a priority for them to attend the training. We initiated just one workshop - for Research Nurses, which was relatively well attended, so the training course initiation and attendance was partially feasible.

Attendees had varied levels of experience recruiting into RCTs ranging from less than 1 year to 5 years. In addition to this, five members of staff also stated that they accessed the online module; three attended the workshop, and two who did not. One of the two people who accessed the online course but had not attended the face-to-face training was in the control group.

On a scale of 0 (very poor) to 10 (excellent), participants who attended the workshop felt positively about the format of the course with a mean score of 9.3 (SD 1.0). Participants felt they “learnt a lot” with a mean score of 9.2 (SD 0.9). They also felt that the workshop would make “a lot of difference” to their future recruitment practices, with a mean score of 8.7 (SD 0.9).

### Impact of recruitment training on self-confidence and awareness

At baseline the recruiting staff in the control group seemed to respond more positively (i.e., have more confidence, were more comfortable) to all the self-confidence questions than those in the intervention group. The highest levels of confidence were reported for discussing recruitment to RCTs (on a 10-point scale, where 10 is ‘very confident: mean of 8.2 for control and 7.2 for intervention) and explaining randomisation (mean scores of 8.0 for control and 7.2 for intervention). Table 3 presents the baseline, post-training and follow-up scores for questions assessing self-confidence and awareness.

**Table 3.** Baseline, post-training and follow-up self-confidence and awareness scores reported by recruiters.

Question	Time point	Control	Intervention	Adjusted between-groups difference (95%CI)*	*p*-value
How confident are you about discussing recruitment to RCTs with patients?	Baseline Post-training Follow-up	8.2 (1.5) - 8.3 (1.5)	7.2 (1.8) 8.4 (0.3) 8.3 (1.3)	1.06 (0.25–1.87)	0.01
How easy do you find describing randomisation?	Baseline Follow-up	8.0 (1.6) 8.0 (1.4)	7.2 (1.7) 8.2 (1.0)	0.69 (−0.12–1.51)	0.09
How comfortable are you with explaining uncertainty about the best treatment to patients (i.e., clinical equipoise)?	Baseline Follow-up	7.6 (1.6) 7.6 (1.5)	6.4 (1.6) 8.1 (1.5)	1.16 (0.19–2.13)	0.02
How confident are you about providing complex information about RCTs to highly intelligent patients?	Baseline Follow-up	7.4 (1.6) 7.5 (1.4)	5.4 (1.7) 7.7 (1.3)	1.15 (0.36–1.94)	0.01
How confident are you about providing complex information about RCTs to patients with limited capacity to understand?	Baseline Follow-up	6.8 (2.1) 6.3 (2.4)	6.1 (1.9) 7.6 (1.5)	1.32 (0.21–2.44)	0.02
How comfortable are you with entering patients into RCTs that compare surgery with no surgery/other option?	Baseline Follow-up	6.9 (1.9) 7.7 (1.6)	5.8 (2.9) 7.5 (1.7)	0.19 (−0.82–1.19)	0.71
How confident are you in dealing with the ‘internet guru’ patient?	Baseline Follow-up	6.5 (2.4) 7.5 (1.4)	5.4 (2.1) 7.2 (1.8)	−0.06 (−1.25 to 1.13)	0.92
How confident are you in obtaining authentic informed consent for randomisation from patients who have a deferential attitude towards you?	Baseline Follow-up	6.6 (2.3) 7.4 (1.8)	6.0 (1.7) 7.4 (1.5)	0.69 (−0.13–1.52)	0.10
How confident are you when discussing RCTs with patients who are mistrustful and suspicious about trials and experiments in medicine?	Baseline Follow-up	6.8 (2.2) 7.2 (1.7)	5.6 (2.4) 7.3 (1.7)	0.91 (−0.04–1.86)	0.06
How aware are you of the challenges to trial recruitment?	Baseline Post-training Follow-up	7.5 (2.1) - 8.4 (1.5)	7.5 (1.7) 7.9 (0.5) 8.8 (1.0)	0.92 (0.03–1.80)	0.04
How confident do you feel about overcoming the challenges of RCT recruitment?	BaselinePost-training Follow-up	7.0 (2.1) - 7.7 (1.4)	6.3 (1.7) 8.3 (0.2) 8.3 (1.0)	0.97 (0.15–1.80)	0.02

Mean (SD); Baseline *n*: 22 control, 13 intervention; Post training *n*: 11 intervention; Follow-up *n*: 21 control, 16 intervention.

* This is follow-up adjusted for baseline.

When assessing pre-post scores in the intervention group only (Table 3), participants rated their confidence in discussing recruitment higher after the workshop, with an increase in mean score of 1.45 (95% CI 0.70 to 2.21, *p* = 0.002). They were also more confident about overcoming recruitment challenges with an increase in the mean score of 2.18 (95% CI 1.19 to 3.17, *p* = 0.001). However, the change in their awareness of the recruitment challenges was not statistically significant with an increase in score of 0.45 (95% CI -1.06 to 1.97, *p*= 0.52).

There were 37 responses to the follow-up questionnaire – 21 in the control group (56.8%) and 16 (43.2%) in the intervention group. Thirty participants completed the self-confidence questions at both baseline and follow-up and were included in this analysis (Table 3). The results show that participants in the intervention arm were more confident in: discussing recruitment, explaining the uncertainty of the best treatment, providing complex information to intelligent patients, providing complex information to patients with limited capacity, awareness of the challenges of recruitment and overcoming recruitment challenges. There was no evidence of a difference in confidence in: describing randomisation, entering patients into a trial comparing surgery with no surgery, dealing with ‘internet guru’ patients, gaining informed consent from those with differential attitudes towards them and discussing RCTs with those who mistrust/are suspicious about trials and experiments in medicine.

### Impact of training on screening and recruitment rates

As shown in Table 4, at baseline the numbers of patients approached and/or recruited were similar across both groups, with the most common response being that 1–3 patients were approached and/or recruited on a participant defined typical week in the past month. The number of patients approached in a typical week in the past month at follow-up remained the same as at baseline; this was the same in both groups.

**Table 4.** Number of patients approached and agreeing to participate at baseline and follow-up, as reported by recruiters per participant defined typical week in the last month.

Summary	Control		Intervention		Total	
	Baseline (*n*=22)	Follow-up (*n*=21)	Baseline (*n*=13)	Follow-up (*n*=16)	Baseline (*n*=35)	Follow-up (*n*=37)
Approached						
0	2 (9.1)	9 (42.9)	0 (0.0)	7 (43.8)	2 (5.7)	16 (43.2)
1–3	11 (50.0)	1 (4.8)	8 (61.5)	0 (0.0)	19 (54.3)	1 (2.7)
4–6	5 (22.7)	5 (23.8)	5 (38.5)	5 (31.2)	19 (54.3)	10 (27.0)
7–10	3 (13.6)	5 (23.8)	0 (0.0)	2 (12.5)	3 (8.6)	7 (18.9)
>10	1 (4.6)	0 (0.0)	0 (0.0)	0 (0.0)	1 (2.9)	0 (0.0)
Missing	0 (0.0)	1 (4.8)	0 (0.0)	2 (12.5)	0 (0.0)	3 (8.1)
**Recruited**						
0	3 (13.6)	0 (0.0)	1 (7.7)	1 (6.3)	4 (11.4)	1 (2.7)
1–3	12 (54.6)	11 (52.4)	8 (61.5)	9 (56.3)	20 (57.1)	20 (54.1)
4–6	6 (27.3)	6 (28.6)	3 (23.1)	3 (18.8)	9 (25.7)	9 (24.3)
7–10	1 (4.6)	3 (14.3)	0 (0.0)	1 (6.3)	1 (2.9)	4 (10.8)
>10	0 (0.0)	0 (0.0)	1 (7.7)	0 (0.0)	1 (2.9)	0 (0.0)
Missing	0 (0.0)	1 (4.8)	0 (0.0)	2 (12.5)	0 (0.0)	3 (8.1)

Data were collected on screening, eligibility and recruitment by all sites across a defined 6 month period. Twenty three sites were included in the analysis (15 PROFHER-2, 5 IntAct, and 3 START:REACTS; 13 intervention, 11 control); data from the DISC trial was excluded as the timing of screening, eligibility assessment and recruitment was not clearly noted and so data could not be split into pre- and post-training data. On average, 0.69 recruiting staff from each intervention site attended the training – six sites had no one attend training (46.2% of the intervention sites in the analysis); and two sites had two members of staff attend.

On average, 6.9 patients were screened over the 6 months post-training; 7.0 in the intervention group and 6.8 in the control group. The number screened differed between the host trials, with an average of 4.8 in PROFHER-2, 10.4 in IntAct and 11.7 in START:REACTS. There was no evidence of a difference in screening between sites randomised to the intervention versus sites allocated to control (coefficient −0.35, 95% CI -7.84 to 7.15, *p* = 0.92). Results were maintained when the model was re-run to include screening in the month immediately before training (coefficient 1.50, 95% CI -5.44 to 8.44, *p* = 0.66).

Over the six months post-training, the average eligible to recruited conversion rate across the participating studies was 58%; it was 55% in the intervention group and 63% in the control group. This also varied by the host trial; there was an average of 34% in PROFHER-2, an average of 51% in START:REACTS, and IntAct had 100% eligible patients recruited for all 6 months post-training. Linear regression, adjusting for host trial and SWAT allocation, for the 15 sites (63%) with screening activity post-training identified there was no evidence of a difference in recruitment rate (coefficient −0.07, 95% CI -0.43 to 0.29, *p* = 0.66), and the findings were maintained when the model was re-run to include screening in the month immediately before training (coefficient − 0.08, 95% CI -0.45 to 0.29, *p* = 0.64).

## Discussion

### Summary of main findings

We have demonstrated that it is feasible to recruit multiple surgical host trials to undertake a coordinated SWAT simultaneously to evaluate the effectiveness of a staff training course. There were however, important differences between the staff groups in terms of enrolment to the training. Some nurses and allied health professionals attended the training course, although this was only 11 of 29 who expressed an interest amongst the intervention sites; less than one per intervention site. As the surgeon workshop had to be cancelled, a key cohort of recruiting staff, often with a central role in study and treatment discussions, were not represented. As a result, the true effect of the training may be impacted, and so further evaluations are therefore required to assess the training. Given the low engagement observed in this SWAT, the future success of evaluating RCT training interventions using SWAT methodology will require looking at how to improve identification and engagement of core recruiting staff, and to employ strategies to increase attendance at training.

Mills et al^16^ saw 67 surgeons attending similar intervention training with a cap needing to be put on attendance. Mills et al suggest that this engagement was largely because of personal invitation and encouragement by the study Chief Investigator of each trial to attend, something which was not maximised in this study. Restricted dates or location did not appear to be an issue in the previous study as the surgeons did not always choose dates for training, and the location was less central than in the present study (South West England, as opposed to the Midlands). Given clinical commitments, a long lead-in time for training may have been helpful to ensure that clinicians could plan for training with sufficient time to change clinical duties. Future studies of training interventions should therefore consider who is best placed to approach, invite and encourage trial members to attend similar events, and should consider appropriate lead times to allow rescheduling of clinical responsibilities.

To supplement learning, access to the GRANULE online e-learning course was provided to intervention participants following the face-to-face training course, or in place of if staff were unable to attend. While this online training presents a reduced version of the face-to-face course, focusing primarily on the key hidden challenges with recruitment discussion, this or other similar online courses may afford an opportunity to maximise attendance by removing the need to travel. Given the increasing use of remote, video conferencing meeting and training technologies arising from the COVID-19 pandemic, this is perhaps now even more pertinent and therefore the medium by which training is delivered should be an important consideration for future studies.

We were able to collect data on screening and recruitment rates from three of the four trials and found that there was no evidence of a difference in the numbers of patients screened between the intervention and control groups. Equally, there was no evidence of a difference in the numbers of eligible patients recruited and enrolled into the host trials between the two groups. This may be due to the fact that training was not undertaken with key recruiting staff (i.e., surgeons) and not all intervention staff (i.e., nurses) attended the training, and that and that not all sites enrolled were actively recruiting. The three host trials do however have low screening rates – an average of 1.15 patients per site per month – so it is possible that the prevalence of the conditions being treated in the host trials was too low to see any effect. Additionally, approximately a third of the sites did not screen any patients over the 6 months post training, although it is not clear why this was the case. The recruitment conversion rate of eligible patients for IntAct being 100% throughout however suggests that perhaps not all potential patients were being screened, only those likely to be eligible, which may have influenced the results.

### Strengths and limitations of the study

This SWAT assesses the feasibility of a simultaneous evaluation of a recruitment strategy across four host trials. We were able to use the same SWAT protocol in a standardised way across the different host trials and patient populations. This simultaneous SWAT is also much more efficient than undertaking four separate SWATs, requiring just one regulatory approval for all the SWATs through one central coordination point, which also reduced burden on host trial teams. The simultaneous SWAT also provided an economy of scale, with one central team instead of multiple host trial teams planning, undertaking, analysing, and reporting the SWAT. Undertaking evaluation across multiple host trials simultaneously can significantly speed up generating the evidence-base for recruiting participants into trials; and, consequently, improving trial efficiency and reducing research waste. However, this is only the case if key personnel attend training. We found it challenging to engage surgeons to attend the face-to-face training, with only one surgeon being available to attend the training. As a result, a key cohort of recruiting staff were not represented which may limit the findings here. Delivering the SWAT simultaneously in four host trials limited our ability to be flexible with the training dates, which may have made it more difficult for surgeons to have sufficient notice to fit this around their clinical commitments. Future studies should consider delivering the training at a time that fits surgeons’ schedules and potentially involving a surgeon Chief Investigator (CI)/trials leader in the delivery of the intervention. The training venue, whilst central in the UK, may also have impacted on surgeons living in more remote areas of the UK, as additional time would have been required to travel to and from the venue.

To ensure the training occurred within a reasonable timeframe, to give focus to the discussions with host trials, and for resource reasons, we agreed two dates for the training with the training team (one date for Surgeons, and one date for Research Nurses). Whilst having these dates was helpful for some discussions, it also introduced a ‘deadline’ for host trial recruitment, which may have impacted on our ability to recruit further host trials. However, most host trials had sufficient lead time as they were recruited approximately six months prior to the training (with recruiters having at least 3 months’ notice to attend the training), except for one, which was recruited six weeks prior to the training.

Recruiting host trials for this simultaneous SWAT required significant logistical coordination and demanded a central coordination point. The process for recruiting host trial teams typically involved an initial approach to the CI. If the CI was interested, then they would discuss the SWAT with their team to obtain buy-in, including from the TMG and Trial Manager. If there was buy-in, then there were further discussions about how the SWAT would align with the host trial, how sites would be approached, and about other logistical and methodological issues. This alignment was not always possible, and one host trial was not recruited because although interested in the training SWAT, they had different ideas about how they wanted the SWAT to be delivered in their trial. For instance, they wanted significantly more training dates for both nurses and surgeons than we were able to offer, and wanted outcomes to be followed up over their entire recruitment phase of 3 years, which we were unable to accommodate for resource reasons.

The GRANULE training provided to intervention participants was published and freely and widely available by the time of the training and follow-up period of the SWAT. To avoid contamination in the control sites having access to training based on the same principles as the training delivered as part of the SWAT, we asked the host trial teams not to promote the GRANULE training to their sites during the follow-up period of the SWAT. At each follow-up time point, we asked all participants whether they had accessed the GRANULE training. Only three recruiters in the intervention group reported accessing the online training, and one participant in the control arm also reported accessing the training online. This was not therefore a resource well-utilised by recruiters in the intervention group, and although there was contamination, this was to a limited extent.

Screening and recruitment rates were available for three of the four trials, with data from one trial being omitted from analyses as the way in which data were routinely collected meant data could not be split into pre- and post-training time periods. This therefore impacts the assessment of training intervention effectiveness on screening and recruitment and so future SWATs should consider streamlining data collection to ensure all data can be included.

The poor baseline data completion in the intervention group accounted for the higher levels of missing data in the intervention group, and may have accounted for the differences observed in baseline characteristics between the intervention and control groups. Whilst we reported the number of participants with missing baseline by allocation, the SWAT might have benefited from a sensitivity analysis with respect to missing data. However, the surgeons who did not attend the training also completed baseline and outcome data. The statistical analysis also adjusted for baseline score, and we believe this imbalance was due to chance, despite our efforts to protect against it.^24^ This chance bias may have influenced the outcomes observed.

### Comparison with existing literature

Our study builds on the work of the MRC-funded Systematic Techniques for Assisting Recruitment to Trials (START) programme, which established the feasibility of undertaking evaluations of the same recruitment strategies across multiple host trials.^25, 26^ These evaluations occurred in individual host trials at different times, depending on the host trial’s timescale.

Our findings are consistent with the observational study that evaluated earlier versions of this recruitment training course delivered to 67 surgeons and 32 research nurses who between them were recruiting to over 40 surgical RCTs. In both studies, the training was found to improve the confidence of recruiting staff, both immediately after and for up to three months following the training, with consistency across the two studies regarding improvement of individual confidence aspects.^16^

Similarly, a systematic review of studies across any clinical area that evaluated training programmes for trial recruiters found the training programmes were well received and some increased recruiters' self-confidence in communicating key RCT concepts to patients. However, this review found little evidence that this training increased actual recruitment rates or patient understanding, satisfaction, or levels of informed consent.^13^ Another systematic review of the effectiveness of education and training interventions for recruiters to trials which included both randomised and non-randomised trials of any type of education and training intervention for recruiters to trials, within any health care field found three studies that reported recruitment rates.^14^ One study favoured the training intervention for increased recruitment; the other two found no differences between the groups. Quality of informed consent was improved, but no differences between groups in understanding or knowledge of trial information were found. Our study corresponds to the majority of findings from these studies that there was no evidence of a difference in the numbers of patients screened, or eligible patients recruited, between the intervention and control groups.

Authors JD, NM, LR, MJ, and JB developed the QuinteT Recruitment Intervention (QRI), which includes a bespoke training programme tailored for individual trials as part of the intevention.^27^ There are a number of advantages to tailored training. Firstly, problems that are unique to the host trial can be elicited as they occur and addressed using context specific solutions, which is difficult to do in a generic training context. Secondly, the tailored training can be timed to the needs of the host trial and individual recruiters within it. There might be more engagement with recruiters as they might see the trainers as ‘part of the team’, and therefore the training is seen to be more ‘relevant’.

Generic training can be scaled up in a way that is not possible for tailored training. This can include delivery of the training using online and e-learning platforms. This has the potential to reach many more trial teams and more recruiting staff more quickly, which may be a cost-effective way of up-skilling trial recruiters.

### Implications for future research

There is a need for further robust evaluation of recruitment training interventions for staff using SWAT methods, drawing on the learnings of this initial feasibility study. Future SWATs may wish to consider including recruiter training, as part of a wider randomised, recruitment intervention.

Recruitment is a team process and so it is essential that all those involved in this task must engage appropriately with training and relevant support. The non-completion of baseline data rate was high overall and impacted on the robustness of the SWAT and its conclusions. If replicated in a full-scale SWAT, then it would be important to try to reduce the non-completion of questionnaires, particularly at baseline.

Given the lack of engagement of surgeons in this SWAT, future SWATs should consider alternative methods to engage surgeons with training. The previous evaluation of this type of intervention suggests engagement and enthusiasm of Chief Investigators is crucial to support and promote attendance.^16^ Planning training with sufficient time to accommodate clinical commitments is also important, as was considered a key to success in the Mills et al^16^ study . It may also be worth considering the offer of multiple training dates, so that surgeons and other participants can flexibly select the date to attend that best suits them, or to hold training in multiple locations or online (recognising the limitations of engagement), to minimise the travel and time commitment required by participants.

Adopting qualitative methods to explore recruiters’ and patients’ perspectives of recruitment interventions may help to obtain further insights into findings in future evaluations of the training interventions. Guidance on the process evaluation of complex interventions have been published which could aid this accordingly.^28^

Findings from this research will be sent to the authors of the Cochrane systematic review of interventions to improve recruitment to trials, for inclusion in future systematic reviews.

## Conclusions

We have demonstrated the feasibility of testing the same recruitment training intervention across multiple ongoing host trials simultaneously. We were able to enrol recruiting nurses and allied health professionals to the SWAT and follow most of them up, but were unable to deliver the training to surgeons.

The training was acceptable to recruiters that attended; and it improved the confidence of recruiting staff in their perceived ability to recruit patients to the host trial, both immediately after and for up to three months following the training. This therefore demonstrates that there is potential to increase the scope of SWATs from those which are relatively minor procedural changes to complex interventions.

Future SWATs should consider the best methods to engage surgeons with training, drawing on examples from previous successful studies. Studies should also consider technological advances which may facilitate improved attendance given the increase in use due to the Covid-19 pandemic.

## ORCID iDs

Adwoa Parker https://orcid.org/0000-0002-2880-3935

Catherine Arundel https://orcid.org/0000-0003-0512-4339

Nicola Mills https://orcid.org/0000-0002-2960-2940

Leila Rooshenas https://orcid.org/0000-0002-6166-6055

Marcus Jepson https://orcid.org/0000-0003-3261-1626

Jenny L Donovan https://orcid.org/0000-0002-6488-5472

Jane M Blazeby https://orcid.org/0000-0002-3354-3330

Elizabeth Coleman https://orcid.org/0000-0003-4210-1865

Laura Clark https://orcid.org/0000-0001-9227-5447

Laura Doherty https://orcid.org/0000-0001-5310-5808

Catherine E Hewitt https://orcid.org/0000-0002-0415-3536

Prasanna Partha Sarathy https://orcid.org/0000-0002-0221-1771

David Beard https://orcid.org/0000-0001-7884-6389

Peter Bower https://orcid.org/0000-0001-9558-3349

Stephen Brealey https://orcid.org/0000-0001-9749-7014

Paul Brocklehurst https://orcid.org/0000-0003-1878-9030

Julie Croft https://orcid.org/0000-0001-7586-3394

Lucy Culliford https://orcid.org/0000-0002-9255-6617

Joseph Dias https://orcid.org/0000-0001-5360-4543

Declan Devane https://orcid.org/0000-0002-9393-7075

Sandra Eldridge https://orcid.org/0000-0001-5638-2317

Richard Emsley https://orcid.org/0000-0002-1218-675X

Sandra Galvin https://orcid.org/0000-0003-3923-2506

Elke Gemperle-Mannion https://orcid.org/0000-0002-6116-2420

David G Jayne https://orcid.org/0000-0002-8725-3283

Andrew J Metcalfe https://orcid.org/0000-0002-4515-8202

Alan Montgomery https://orcid.org/0000-0003-0450-1606

Amar Rangan https://orcid.org/0000-0002-5452-8578

Christopher J Sutton https://orcid.org/0000-0002-6406-1318

Puvanendran Tharmanathan https://orcid.org/0000-0001-9196-0207

Shaun Treweek https://orcid.org/0000-0002-7239-7241

David Torgerson https://orcid.org/0000-0002-1667-4275
